# Beyond Mendelian randomization: how to interpret evidence of shared genetic predictors

**DOI:** 10.1016/j.jclinepi.2015.08.001

**Published:** 2016-01

**Authors:** Stephen Burgess, Adam S. Butterworth, John R. Thompson

**Affiliations:** a Cardiovascular Epidemiology Unit, Department of Public Health and Primary Care, 2 Worts Causeway, Cambridge CB1 8RN, UK; b Homerton College, University of Cambridge, Hills Road, Cambridge CB2 8PH, UK; c Department of Health Sciences, Adrian Building, University Road, Leicester, LE1 7RH, UK

**Keywords:** Mendelian randomization, Instrumental variable, Causal inference, Genetic variants, Genetic predictors, Aetiology, Translational Genetics

## Abstract

**Objective:**

Mendelian randomization is a popular technique for assessing and estimating the causal effects of risk factors. If genetic variants which are instrumental variables for a risk factor are shown to be additionally associated with a disease outcome, then the risk factor is a cause of the disease. However, in many cases, the instrumental variable assumptions are not plausible, or are in doubt. In this paper, we provide a theoretical classification of scenarios in which a causal conclusion is justified or not justified, and discuss the interpretation of causal effect estimates.

**Results:**

A list of guidelines based on the ‘Bradford Hill criteria’ for judging the plausibility of a causal finding from an applied Mendelian randomization study is provided. We also give a framework for performing and interpreting investigations performed in the style of Mendelian randomization, but where the choice of genetic variants is statistically, rather than biologically motivated. Such analyses should not be assigned the same evidential weight as a Mendelian randomization investigation.

**Conclusion:**

We discuss the role of such investigations (in the style of Mendelian randomization), and what they add to our understanding of potential causal mechanisms. If the genetic variants are selected solely according to statistical criteria, and the biological roles of genetic variants are not investigated, this may be little more than what can be learned from a well-designed classical observational study.

## 1 Introduction

**What is new?**

• The popularity of Mendelian randomization as a tool for investigating causal relationships in observational data is currently increasing.
• There is a distinction between Mendelian randomization as it was initially conceived and performed (mainly for circulating biomarkers using few genetic variants in relevant gene regions) and how it is often used today (often opportunistically using large numbers of genetic variants whose functional relevance is unknown).
• Biological guidelines based on the Bradford Hill criteria, and statistical criteria based on associations with measured covariates and homogeneity of evidence across genetic variants are given to help judge the plausibility of a causal conclusion.
• Causal claims should be reserved to cases where the evidence for the instrumental variable assumptions is strong; otherwise the language of common genetic predictors should be used.

Genome-wide association studies have revealed genetic predictors of many clinically relevant traits, including modifiable risk factors and disease outcomes. Many investigators have taken two such traits and considered the statistical question of whether genetic variants that are associated with trait A (often taken to be a risk factor and viewed as a putative cause) also show an association with trait B (often taken to be a disease outcome), for example, under the heading of Mendelian randomization [1], [2]. However, conclusions from such analyses have been diverse, ranging from a direct causal interpretation (trait A causes trait B) to one of shared etiology (trait A and trait B have common predictors). In this article, we consider conditions under which a causal interpretation is justified and discuss situations in which weaker conclusions are more appropriate.

## 2 Classification of scenarios

We consider the following classification of possible scenarios for the relationship between two variables A and B such that genetic variant(s) associated with A are also associated with B. An interventional definition of causality is presumed; A is a cause of B means that intervention on the distribution of A results in changes to the distribution of B [3]. We assume that either logic or biological knowledge is able to provide an ordering between A and B by which A is the putative cause and B is the putative effect. The three scenarios we consider are as follows:

1. A is a cause of B, and all causal pathways from the genetic variant(s) to B pass through A;
2. A is a cause of B, but there are alternative causal pathways leading from the genetic variant(s) to B which do not pass through A;
3. A is not a cause of B—the genetic variant(s) are independently associated with A and B.

**Fig. 1:**
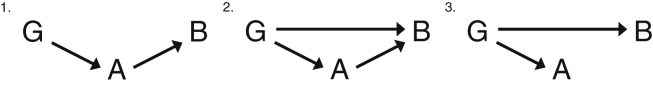
Diagrams illustrating scenarios of causal relationships between selected genetic variant(s) G, putative causal trait A, and putative effect trait B, compatible with genetic variant(s) being associated with both traits.

Diagrams representing the relationships between the variables in each case are given in Fig. 1. We continue to explore each of the scenarios mentioned previously in turn.

### 2.1 All causal pathways through risk factor

To infer a causal effect of A on B, it is necessary that genetic variants used in the analysis satisfy the assumptions of an instrumental variable [4], [5]:

1. The set of genetic variants is associated with the risk factor A;
2. Each genetic variant is independent of confounders of the association between A and B;
3. If the risk factors were kept constant, intervention on the genetic variant(s) would not have an effect on the outcome.

**Fig. 2:**
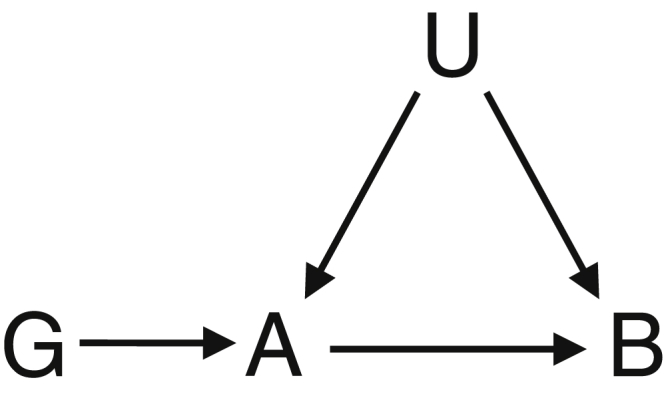
Diagram illustrating causal relationships between genetic variant(s) G, putative causal trait (risk factor) A, putative effect trait (outcome) B, and confounders U necessary for instrumental variable assumptions to be satisfied.

A diagram corresponding to these assumptions is given in Fig. 2.

**Fig. 3:**
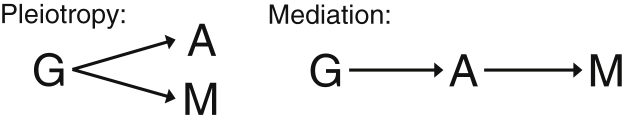
Diagrams illustrating the difference between pleiotropy (left), where genetic variant G is independently associated with traits A and M, and mediation (right), where G is associated with trait M only via the effect of A.

These assumptions imply that all causal pathways from the genetic variant(s) to the outcome pass through the putative causal risk factor, and there are no alternative pathways not via the risk factor [6]. Formal considerations about how causal pathways are defined are given in the Appendix at www.jclinepi.com. The assumptions require that genetic variants used for the assessment of the causal nature of a risk factor must be specific in their associations with the risk factor, although they may also show associations with other variables via downstream effects of the risk factor. For example, genetic variants that are candidate instrumental variables for body mass index (BMI) may show associations with C-reactive protein (CRP), because of a causal effect of BMI on CRP [7]. This means that the genetic variants can have associations with other variables via mediation (ie, the genetic association with the other variable is mediated via the risk factor of interest), but not via pleiotropy (ie, the genetic association with the other variable is via a different causal pathway and not via the risk factor of interest; Fig. 3).

If we seek to assess whether there is a causal effect of A on B, but not to provide an estimate of a causal effect parameter, then only the three instrumental variable assumptions listed previously are required. Under these assumptions, an association between the outcome B and genetic variants which are instrumental variables for A implies a causal effect of A on B [8]. To estimate a causal effect parameter, further assumptions are required [9], including linearity of the risk factor–outcome association, and the stable unit treatment value assumption (the value of the outcome for each individual depends on the value of the risk factor, and not on the mechanism by which the risk factor was intervened on) [10].

Mendelian randomization can be understood as being similar to a randomized controlled trial, in which genetic variants play the role of random assignment to a treatment group [11]. However, in a randomized trial, the goal is to assess the effect of different treatment strategies between randomized subgroups with the purpose of implementing one of the strategies, whereas in Mendelian randomization, the goal is to assess the effect of a difference in the distributions of a risk factor between genetically determined subgroups, with the purpose of implementing a nongenetic intervention on the risk factor. The genetically determined differences in the risk factor are likely to differ from any proposed intervention on the risk factor in a number of qualitative and quantitative ways: in particular because of the duration of the intervention (life long or short term), the magnitude of the intervention (genetic effects are usually small, clinical interventions are typically larger), and the mechanism of the intervention (genetic effects and clinical interventions may operate via different pathways) [12]. As different ways (including timing, duration, mechanism, and magnitude) of intervening on the risk factor will typically lead to different magnitudes of effect on the outcome, it is likely that the causal effect estimate from a Mendelian randomization study differs quantitatively from the effect of a proposed intervention in the risk factor. Hence, even in a scenario in which the genetic variant(s) are valid instrumental variables and the risk factor-outcome relationship is linear, a causal effect estimate from a Mendelian randomization study should not be interpreted literally as the expected outcome of an intervention on the risk factor of interest.

For this reason, some authors have questioned whether causal effect estimates should ever be presented as part of a Mendelian randomization analysis [13]. Although a causal estimate in a Mendelian randomization study will typically differ from the expected result of a clinical intervention on a risk factor, there are practical reasons why it may be beneficial to provide a causal estimate in a Mendelian randomization study, provided the magnitude of this estimate is not overinterpreted.

• Generally in epidemiology, estimates with confidence intervals are preferred to hypothesis tests with *P*-values, as they are more informative [14]. If a *P*-value does not achieve conventional levels of statistical significance, a point estimate with a confidence interval allows the reader to judge in a quantitative way whether the null result reflects a lack of evidence or a genuine negative finding in comparison with either the observational association, or with a minimal clinically relevant effect. If the confidence intervals for the causal effect exclude the minimal clinically relevant effect, then the causal effect for all practical purposes is null, particularly as Mendelian randomization estimates often overestimate the effects of intervening on risk factors in practice (as they represent life-long effects) [12]. Additionally, a magnitude of causal effect must be proposed to perform a formal power calculation [15]. This is particularly important in Mendelian randomization analyses, which often suffer from limited power to detect a causal effect of potential clinical interest [16].
• If several genetic variants are valid instrumental variables for the same risk factor, greater power to detect a clinically relevant causal effect can be obtained using information on all the variants simultaneously rather than that using the variants individually [17]. It may be that no variant individually provides strong evidence for a causal effect of the risk factor based solely on its association with the outcome, but the combination of evidence from all of the variants does. Causal estimates from multiple variants also enable the quantitative comparison of the consistency of genetic associations, using a heterogeneity or overidentification test as a statistical assessment of pleiotropy [18].
• Although a causal estimate from a Mendelian randomization investigation will not correspond precisely to the expected effect of a intervention in the risk factor (which will in any case differ between interventions), it does represent the outcome of a well-defined intervention, namely in the genetic code at conception. As such, it will be a more relevant indicator of the predicted effect of a clinical intervention in the risk factor if the intervention acts in a similar way to the genetic variant; for example, if the genetic variant and the intervention affect the same biological pathway, if the magnitudes of change in the risk factor are similar, and if long-term changes in the risk factor are considered.

In summary, if the only causal pathways from the genetic variant(s) to the outcomes are via the risk factor of interest, then the causal hypothesis of the risk factor on the outcome can be reliably assessed, although a numerical causal estimate will be at best an approximation to the effect of intervening on the risk factor in practice.

### 2.2 Alternative causal pathways not through risk factor

Often, the associations of a genetic variant are not restricted to the risk factor of interest. “Off-target” genetic associations, including pleiotropic effects and associations arising from linkage disequilibrium, may lead to violation of the instrumental variable assumptions by providing an alternative pathway from the genetic variant(s) to the outcome not via the risk factor [6]. If a genetic variant violates the instrumental variable assumptions, then any assessment of causality using that variant will be unreliable [19], [20].

**Fig. 4:**
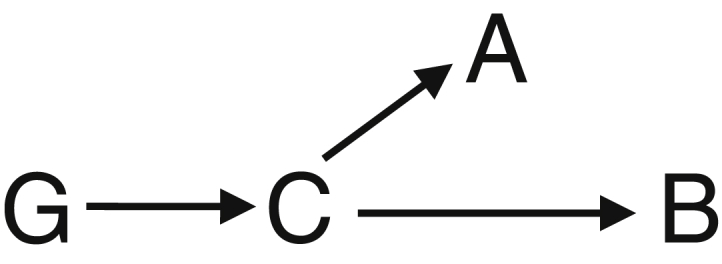
Diagram illustrating additional scenario of causal relationships between selected genetic variant(s) G, underlying putative causal trait C, measured proxy variable A, and putative effect trait B, compatible with genetic variant(s) being associated with both traits A and B (confounding variables are omitted from the diagram).

We consider an alternative set of assumptions (scenario 2a, Fig. 4) under which there may be an alternative causal pathway from the genetic variant(s) to the outcome not via the risk factor, but testing the genetic associations with the outcome still provides a valid test of the null hypothesis of no causal relationship. In this case, we assume that the effect of the genetic variant(s) is via an underlying causal variable C, and the measured risk factor is a surrogate (or proxy) measure of the underlying causal variable(s). For example, BMI can be used as a surrogate measure of obesity. Provided that all the genetic variants used in a Mendelian randomization analysis are exclusively associated with some aspect of obesity that is captured by BMI, associations of the genetic variants with the outcome are indicative of a causal role of obesity in disease risk. However, unless a specific causal risk factor can be identified such that all causal pathways from gene to disease run via that risk factor, no more detailed causal claim can be made [21]. In particular, any causal effect estimate will be an even more distant approximation of the potential result of intervening on the risk factor in practice.

Another example of scenario 2a, where the instrumental variable assumptions are not formally satisfied, but a Mendelian randomization analysis may be informative, involves genetic variants associated with smoking. The associations with lung cancer of certain genetic variants related to smoking did not appear to be mediated by a measure of smoking intensity, the number of cigarettes smoked per day [22]. A Mendelian randomization estimate expressed as the causal effect of number of cigarettes per day on lung cancer risk using one variant gave an odds ratio estimate of 2180, an implausibly large effect [13]. This could be interpreted as meaning that the instrumental variable assumptions are not satisfied, as there appears to be an alternative causal pathway from the genetic variants to the outcome not via smoking intensity. However, an alternative interpretation would be that smoking intensity is a proxy measure of the true underlying causal risk factor, but an imprecisely measured proxy, so that the estimate provides a valid test of the causal null hypothesis, but the causal effect is overestimated. The general conclusion that smoking-related behaviors are causally related to lung cancer risk, rather than a specific conclusion about the causal effect of smoking intensity, is more appropriate according to the genetic evidence. A proposal as to the underlying causal risk factor in this case is the amount of nicotine extracted from each cigarette [23]. This scenario is likely to occur for complex exposures that have multiple potential causal pathways.

### 2.3 No causal effect of risk factor

If the instrumental variable assumptions are not satisfied, genetic variants may be associated with a risk factor and an outcome without a causal effect of the risk factor on the outcome. For instance, genetic variants may be associated with a common cause of the risk factor and outcome. Variants in the *IL6R* gene region have been shown to be associated with CRP (an inflammation marker) and with coronary heart disease risk [24], [25]; however, it is thought that interleukin-6 (an upstream marker of inflammation) pathways are causal for coronary heart disease and not CRP itself (as focused Mendelian randomization investigations using variants in the CRP gene region have suggested a null causal effect of CRP on coronary heart disease risk [26]). If variants in the *IL6R* gene region were assumed to be instrumental variables for CRP, then the false conclusion would be reached that CRP was causal for coronary heart disease risk. Although in this case, the use of variants in the *IL6R* gene region as instrumental variables for CRP would be an elementary mistake, in cases where the causal gene and the biological pathway it affects are not known, misleading conclusions could be reached.

Even if the risk factor would seem logically to take the role of the cause and the outcome of the effect, a reverse causal explanation is possible. For example, although inflammatory biomarkers may be thought of as a potential cause of coronary heart disease, it may also be that subclinical disease leads to elevated levels of the biomarkers [27]. Genetic variants associated with coronary heart disease risk via alternative causal pathways may show associations with inflammatory markers because of a reverse causal effect.

It is also important to appreciate that the causal question addressed by a Mendelian randomization is whether long-term elevated (or reduced) levels of a risk factor will affect the outcome. For example, the null causal effect of CRP on coronary artery disease risk estimated using genetic variants having modest associations with CRP levels [26] suggests that the development of pharmacological agents to suppress usual CRP concentrations is not likely to be effective in reducing coronary heart disease incidence. The causal question about long-term levels of the risk factor is usually the relevant question for epidemiologic research.

Distinguishing scenarios 1 and 2, where the risk factor is a cause of the outcome, from scenario 3, where the two have common genetic predictors but are otherwise independent, is not empirically possible and requires biological knowledge. As such, if the instrumental variable assumptions in a particular applied investigation are uncertain, a more tentative conclusion is appropriate. In practice, the distinction between more plausible Mendelian randomization investigations and less plausible ones will be a subjective assessment and will give a continuous scale of evidential quality rather than a dichotomy of “good” and “bad” studies. In the next section, we consider some criteria to help judge the plausibility or otherwise of a Mendelian randomization investigation.

## 3 Assessing the assumptions necessary for causation

Justification of the instrumental variable assumptions can be provided using biological knowledge and statistical testing. In Table 1, we apply the Bradford Hill criteria for causation to Mendelian randomization as a checklist to judge whether a causal conclusion based on the genetic variant(s) is warranted. Of particular interest is the tension between using large numbers of genetic variants, which allows increased power for the assessment of the consistency of the causal effect and its biological gradient across different variants, and specificity, which suggests that an analysis should be limited to variants in those gene regions that most credibly satisfy the instrumental variable assumptions.

The Bradford Hill criteria also suggest that variants from candidate gene investigations, where the function of the genetic variant(s) is well-understood, will have more credibility for use in Mendelian randomization studies than variants with unknown functional relevance, such as those often discovered in genome-wide association studies. Additionally, the utility for translational research of such an analysis will be increased, as a genetic variant with well-understood biology associated with a causal risk factor often indicates a potential pathway for intervention on the risk factor [32]. For instance, genetic variants in the *HMGCR* and *PCSK9* gene regions associated with low-density lipoprotein cholesterol and coronary heart disease risk suggest that inhibition of 3-hydroxy-3-methylglutaryl-coenzyme A reductase (HMGCR) and of proprotein convertase subtilisin/kexin type 9 (PCSK9) would reduce low-density lipoprotein cholesterol levels and therefore be protective of coronary heart disease risk; the former mechanism is how statin drugs act [33], and the drugs targeting the latter mechanism are already in late-stage development [34], [35].

Although the instrumental variable assumptions cannot be statistically proven, implications of the assumptions can be tested [21]. The associations of genetic variants with measured covariates can be tested. A valid instrumental variable should be associated with the risk factor but not with other covariates unless they are causally downstream of the risk factor. Indeed, if there are variables that are known to be causally related to the risk factor, the genetic associations with these variables can be tested as a “positive control.” The consistency of associations of different genetic variants can be assessed visually, in a graph of the per allele genetic associations with the outcome against the per allele associations with the risk factor for each variant (which, apart from random sampling variation, should be a straight line through the origin), and formally by a heterogeneity test [36] (also known as an overidentification test [37]). Departure from a linear relationship may be an indication of pleiotropy. Any outliers on this graph should be examined closely for pleiotropic associations that might explain the genetic association with the outcome. This is particularly important when an allele score (also known as a gene score or genetic risk score) is used to obtain inferences [38]; the overall association of an allele score with the outcome may conceal inconsistencies in the analysis, such as genetic variants having different directions of association with the outcome. Additionally, a funnel plot can be plotted of the instrumental variable estimate on the basis of each genetic variant in turn against the association of the variant with the risk factor. Asymmetry in the funnel plot would also be evidence of differences between estimates from weaker and stronger genetic variants, another possible indication of pleiotropy [39].

## 4 Joint association studies

Recently, several investigators have considered genetic variants associated with a risk factor and their association with an outcome variable in the absence of biological knowledge about the genetic variants. For example, investigators have taken all the genetic variants associated with height at a genome-wide level of statistical significance and considered whether these variants also predict colorectal cancer risk [40]. Although it is impossible to ever have complete biological knowledge about genetic variants to justify the instrumental variable assumptions in a Mendelian randomization investigation, in these studies, the choice of genetic variants is primarily motivated by statistical rather than biological considerations. The instrumental variable assumptions are investigated in a post-hoc way, if at all. We assert that these investigations, although they may use the statistical methodology of Mendelian randomization, are not true Mendelian randomization investigations.

To distinguish these analyses from well-justified Mendelian randomization analyses, we use the term “joint association study” as the joint association of variants with a risk factor and an outcome is assessed. A non-null finding from a joint association study will still provide suggestive evidence of a causal effect, or of shared causal mechanisms, through the conclusion of common genetic predictors [41]. Although a joint association study is not able to assess a causal relationship in a reliable way, a relevant practical question is how to perform and interpret such an analysis so as to provide the best possible evidence for causal inference.

## 5 Performing a joint association study

If there is insufficient biological knowledge to justify the instrumental variable assumptions for a set of candidate genetic variants, several approaches for the selection of variants can be taken as follows.

### 5.1 Conservative approach

If the instrumental variable assumptions can be justified or are more plausible for a subset of variants, then the primary analysis should be based on these variants, and a more speculative analysis using more variants (but potentially having greater power) should be viewed as a secondary analysis.

### 5.2 Liberal approach

If the instrumental variable assumptions cannot be justified biologically, an analysis can be performed using those variants which are associated with the risk factor of interest, but not associated with measured covariates which are potential confounders. Although this approach does not address the difficulty of unknown and unmeasured confounders, sensitivity analyses can be performed to give a sense as to how robust the finding is to violation of the instrumental variable assumptions [42].

### 5.3 Data-driven (post hoc) approach

Alternatively, a data-driven approach has been proposed on the basis of a heterogeneity test statistic for the causal effect estimates from multiple genetic variants [36]. If the statistic exceeds a critical value of the chi-squared distribution (say, the 95th percentile), then a stepwise selection procedure can be followed, omitting the variant whose contribution to the heterogeneity statistic is the greatest, until the statistic is below the critical value [43].

There are several potential pitfalls with such an approach. First, it is necessary to assume that most genetic variants do satisfy the instrumental variable assumptions, so that the outlying variants removed in the stepwise selection are the invalid variants. Second, even if all genetic variants are valid instrumental variables, some heterogeneity in their associations with the outcome would be expected, particularly if the genetic variants influenced the same risk factor via different causal pathways. Third, as with all post-hoc analyses in which the analysis method is determined on the basis of the observed data, there is likely to be bias in the effect estimates. If associations of most genetic variants lie on a straight line with a limited number of rogue variants, then a post-hoc analysis may be a reasonable sensitivity analysis. If there is considerable heterogeneity between variants with no discernible pattern of association, then results from a data-driven analysis will not be reliable.

### 5.4 Whole-genome approach

A final alternative is a whole-genome approach, where genetic variants from throughout the genome are included in an analysis. It has also been suggested that investigations with large numbers of genetic variants may be fruitful [44]; however, there is no strong theoretical justification for this. An analysis using genome-wide genetic scores for various risk factors gave false negative and false positive results; it suggested that CRP was a causal risk factor for coronary heart disease risk (*P* = 0.028), but BMI was not (*P* = 0.37) and suggested an inverse effect of low-density lipoprotein cholesterol on hypertension (*P* = 0.011) and on type 1 diabetes risk (*P* = 0.018) [45]. A more recent analysis using publicly available summarized data on genetic associations using a novel methodologic approach gave more plausible results, although again showed weak to null associations for the established risk factors of obesity and low-density lipoprotein cholesterol with coronary artery disease risk [46].

## 6 Interpreting a joint association study

If genetic variants associated with two traits overlap, this increases the likelihood that the traits have related biological mechanisms. For example, genetic approaches have been used in the nosology of psychiatric disorders to inform the degree to which separately classified diseases may be related [47]. Here the aim was not to assess a particular causal risk factor, but to increase or decrease the general plausibility that similar causal mechanisms underpin each of the disease traits. A genetic approach to assessing the relatedness of two traits is likely to give mechanistic insights beyond what can be obtained from an observational study if common genetic pathways predicting the traits can be identified. Additionally, genetic variants tend not to be associated with socioeconomic or environmental factors, which are difficult to measure in observational research [48]. They are also fixed at conception, so cannot be affected by changes in external variables that may lead to reverse causation in observational studies. Hence, even if the instrumental variable assumptions are not satisfied, a joint association study offers some benefit over an observational analysis in terms of reduced confounding and reverse causation. Finally, genes have functional relevance in biological processes, so shared genetic associations are more likely to represent shared biological rather than nonbiological predictors.

However, the complete separation of biological from nonbiological effects using population genetics is not possible. For example, a genetic variant associated with both alcohol consumption and cannabis use has been interpreted as evidence in the hypothesis that alcohol consumption causes increased cannabis consumption [49]. However, the consequences of having the alcohol-related genetic variant are not limited to biological effects. The decreased propensity to drink alcohol associated with the null form of the genetic variant would also have effects not confined to the biological effect of alcohol consumption. For example, those who do not drink alcohol are also less likely to attend social events at which alcohol is served. The causal effect assessed by a Mendelian randomization experiment in this case is therefore not simply the biological effect of alcohol consumption, but also the social effect of being an alcohol consumer.

Although a joint association analysis can increase or decrease the plausibility of a causal relationship, there are many limitations to such an analysis. This means that the evidential weight of such an analysis in terms of proving or disproving causation should not be as great as that of a Mendelian randomization study in which the instrumental variable assumptions are strongly supported by biological and statistical justification. An analysis where the choice of genetic variants is made solely on the basis of observational data and the biological pathways affected by the variants are not investigated should not carry much more evidential weight for demonstrating a causal relationship than a well-designed classical (nongenetic) observational study.

## 7 Conclusion

Mendelian randomization has been defined as “using genes as instrumental variables for making causal inferences” [2]. As such, if the instrumental variable assumptions are not satisfied, an analysis to demonstrate shared genetic predictors of a risk factor and outcome is not Mendelian randomization, even if the statistical methodology of Mendelian randomization (instrumental variable analysis) is used.

Mendelian randomization has been advocated as providing strong evidence for causal relationships and has been placed in a hierarchy of evidence only below well-designed randomized controlled trials [50]. However, the quality of evidence provided by a Mendelian randomization study relies heavily on the instrumental variable assumptions. Although the finding of shared genetic predictors is consistent with a causal relationship between the risk factor and outcome and increases the plausibility of either the specific risk factor or a mechanism related to the risk factor having a causal effect on the outcome, it is also consistent with the risk factor and outcome simply sharing common predictors. Although speculative “Mendelian randomization” analyses, such as those based on variants of unknown biological relevance discovered in a genome-wide association study, have a role in the scientific literature (as do observational studies and many other designs), results will be far less reliable than those from analyses where the biological role of the genetic variants is well established. Claims of the causal (or noncausal) role of a particular risk factor should be reserved to those where there is strong evidence (biological and statistical) supporting the instrumental variable assumptions, and the weaker claim of common genetic predictors should be made in other cases.

## Figures and Tables

**Table 1:** Bradford Hill criteria applied to Mendelian randomization for judging plausibility of instrumental variable assumptions

The Bradford Hill criteria [28] form a systematic summary of common-sense principles for judging causality that are as relevant in genetic epidemiology as they are in classical epidemiology. We apply each of the relevant criteria to genetic variants for use in Mendelian randomization investigations: **Strength:** If a genetic association with the outcome is slight, then the power of a Mendelian randomization analysis may be low. Additionally, causal estimates are more sensitive to small violations of the instrumental variable assumptions [29]. However, the magnitude of association of a genetic variant with the outcome is not indicative of the importance of that biological pathway in disease risk; if the risk factor can be intervened on by a greater extent than the genetic association (as is often the case with pharmacologic interventions), then a greater impact on the disease outcome may be observed. **Temporality:** As the DNA sequence of an individual is determined at conception, genetic associations are protected from bias due to reverse causation. Genetic variants must always precede the associated variable in time. However, inferring a causal effect of the risk factor on the outcome (rather than the other way round) requires an assumption that the proximal association of the genetic variant is with the risk factor, not with the outcome (nor with an alternative cause of the outcome). **Consistency:** A causal relationship is more plausible if multiple genetic variants associated with the same risk factor are all directionally concordant in their associations with the outcome, especially if the variants are located in different gene regions and/or have different mechanisms of association with the risk factor. **Biological gradient:** Furthermore, a causal relationship is more plausible if the genetic associations with the outcome and with the risk factor for each variant are proportional. For example, genetic associations with low-density lipoprotein cholesterol and with coronary artery disease risk provide evidence of a dose-response relationship, with variants having a greater per allele association with low-density lipoprotein cholesterol also having a greater per allele odds ratio of coronary artery disease [30]. **Specificity:** A causal relationship is more plausible, if the genetic variant(s) are associated with a specific risk factor and outcome and do not have associations with a wide range of covariates and outcomes. A specific association is most likely if the genetic variant(s) are biologically proximal to the risk factor, and not biologically distant. This is most likely for risk factors that are proteins or metabolites (such as C-reactive protein and uric acid), rather than complex risk factors (such as body mass index and blood pressure). **Plausibility:** A causal relationship is more plausible if the function of the genetic variant(s) is known and if the mechanism by which the variant acts is credibly and specifically related to the risk factor. **Coherence:** If an intervention on the risk factor has been performed (eg, if a drug has been developed that acts on the risk factor), associations with intermediate outcomes (covariates) observed in the experimental context should also be present in the genetic context; directionally concordant genetic associations should be observed with the same covariates. For example, associations of genetic variants in the *IL1RN* gene region with C-reactive protein and interleukin-6 should be similar (at least directionally concordant) to those observed in randomized trials of anakinra, the recombinant form of interleukin-1 receptor antagonist [31].
