# Supplementary material for: Beyond Mendelian randomization: how to interpret evidence of shared genetic predictors
Source: J Clin Epidemiol. 2016 Jan;69:208–16. doi: 10.1016/j.jclinepi.2015.08.001 (PMC4687951; doi:10.1016/j.jclinepi.2015.08.001)
Supplement: Appendix [file mmc1.docx]

**Web Appendix**

**Causal pathway**

In the manuscript, in defining an instrumental variable, we stated that there cannot be a causal pathway between the genetic variant(s) and outcome except via the risk factor. Here, we clarify formally what is meant by a "causal pathway".

Formally, the genetic variants and outcome must be *d*-separated by the risk factor and confounders [1]. This means that there cannot be a sequence of edges connecting any genetic variant *G* and the outcome *B* consisting solely of chains (*G → C → B*) or (non-inverted) forks (*G ← D → B*) of variables not including the risk factor. In these examples, *C* may represent a competing risk factor on another pathway, and *D* may represent a selection variable, such as ethnicity, that must be accounted for in the analysis to prevent population stratification [2].

For example, if there is a pathway *G → C → A → B* or *G → A → C → B* (where *A* is the risk factor of interest), then the instrumental variable assumptions are not violated, as the pathway goes through *A*. However, if there is a pathway *G → C*_1_* → C*_2_* → B*, then the instrumental variable assumptions are violated, as there is a pathway from *G* to *B* not via *A*. Equally, if there is a pathway *G ← C*_1_* → C*_2_* → B*, then the instrumental variable assumptions are violated even though the pathway does not consist of arrows pointing in the same direction. However, a pathway such as *G → C*_1_* ← C*_2_* → B* does not consist solely of chains and forks (*C*_1_ is part of an inverted fork), and hence does not violate the instrumental variable assumptions.

**References**

[1] Geiger D, Verma T, Pearl J. Identifying independence in Bayesian networks. *Networks* 1990; **20**(5):507-534, doi:10.1002/net.3230200504.

[2] Lawlor D, Harbord R, Sterne J, Timpson N, Davey Smith G. Mendelian randomization: using genes as instruments for making causal inferences in epidemiology. *Statistics in Medicine* 2008; **27**(8):1133-1163, doi:10.1002/sim.3034.
